# Genome-wide analysis reveals rapid and dynamic changes in miRNA and siRNA sequence and expression during ovule and fiber development in allotetraploid cotton (*Gossypium hirsutum *L.)

**DOI:** 10.1186/gb-2009-10-11-r122

**Published:** 2009-11-04

**Authors:** Mingxiong Pang, Andrew W Woodward, Vikram Agarwal, Xueying Guan, Misook Ha, Vanitharani Ramachandran, Xuemei Chen, Barbara A Triplett, David M Stelly, Z Jeffrey Chen

**Affiliations:** 1 Section of Molecular Cell and Developmental Biology, The University of Texas at Austin, One University Station, A-4800, Austin, TX 78712, USA; 2 Institute for Cellular and Molecular Biology, The University of Texas at Austin, One University Station, A-4800, Austin, TX 78712, USA; 3 Center for Computational Biology and Bioinformatics, The University of Texas at Austin, One University Station, A-4800, Austin, TX 78712, USA; 4 Department of Botany and Plant Sciences, University of California, Riverside, CA 92521, USA; 5 USDA-ARS-SRRC, 1100 Robert E Lee Blvd, New Orleans, LA 70124, USA; 6 Department of Soil and Crop Sciences, Texas A&M University, College Station, TX 77843, USA; 7 Section of Integrative Biology, The University of Texas at Austin, One University Station, A-4800, Austin, TX 78712, USA

## Abstract

**Background:**

Cotton fiber development undergoes rapid and dynamic changes in a single cell type, from fiber initiation, elongation, primary and secondary wall biosynthesis, to fiber maturation. Previous studies showed that cotton genes encoding putative MYB transcription factors and phytohormone responsive factors were induced during early stages of ovule and fiber development. Many of these factors are targets of microRNAs (miRNAs) that mediate target gene regulation by mRNA degradation or translational repression.

**Results:**

Here we sequenced and analyzed over 4 million small RNAs derived from fiber and non-fiber tissues in cotton. The 24-nucleotide small interfering RNAs (siRNAs) were more abundant and highly enriched in ovules and fiber-bearing ovules relative to leaves. A total of 31 miRNA families, including 27 conserved, 4 novel miRNA families and a candidate-novel miRNA, were identified in at least one of the cotton tissues examined. Among 32 miRNA precursors representing 19 unique miRNA families identified, 7 were previously reported, and 25 new miRNA precursors were found in this study. Sequencing, miRNA microarray, and small RNA blot analyses showed a trend of repression of miRNAs, including novel miRNAs, during ovule and fiber development, which correlated with upregulation of several target genes tested. Moreover, 223 targets of cotton miRNAs were predicted from the expressed sequence tags derived from cotton tissues, including ovules and fibers. The cotton miRNAs examined triggered cleavage in the predicted sites of the putative cotton targets in ovules and fibers.

**Conclusions:**

Enrichment of siRNAs in ovules and fibers suggests active small RNA metabolism and chromatin modifications during fiber development, whereas general repression of miRNAs in fibers correlates with upregulation of a dozen validated miRNA targets encoding transcription and phytohormone response factors, including the genes found to be highly expressed in cotton fibers. Rapid and dynamic changes in siRNAs and miRNAs may contribute to ovule and fiber development in allotetraploid cotton.

## Abstract 2

Rapid and dynamic changes in the expression of small RNAs are seen during ovule and fiber development in allotetraploid cotton.

## Background

Cotton fibers are seed trichomes that extend from fertilized ovules. Cotton fiber is among the longest single cells and may grow as long as 6 cm [1]. Cotton fiber cell initiation and elongation are directly affected by plant phytohormones. Auxin and gibberellins are known to promote fiber cell initiation and development [2]. Sequencing analysis of expressed sequence tags (ESTs) from immature ovules and fiber-bearing ovules reveals an enrichment of the transcripts associated with *Auxin Response Factors *(*ARFs*) and gibberellin signaling [3]. Brassinosteroid and ethylene also positively affect fiber development [4,5], whereas abscisic acid and cytokinin inhibit fiber cell development [6]. Moreover, cotton genes encoding putative MYB transcription factors are induced during early stages of fiber development but repressed in a naked seed mutant that is impaired in fiber formation [3,7]. The data agree with the known roles of MYB and other transcription factors in leaf trichome development [8] and cotton fiber development [9,10]. Many genes encoding putative transcription and phytohormone responsive factors are targets of microRNAs (miRNAs).

Small interfering RNAs (siRNAs) and miRNAs are 21- to 24-nucleotide small RNAs produced in diverse species that control gene expression and epigenetic regulation [11-13]. In addition, plants produce *trans*-acting siRNAs (tasiRNAs) [14], stress-induced natural antisense siRNAs (nat-siRNAs) [15], and pathogen-induced long siRNAs [16]. miRNA loci are transcribed by RNA polymerase II into primary miRNA transcripts (pri-miRNAs) that are processed by nuclear RNaseIII-like enzymes such as Dicer and Drosha in animals [17] and DICER-LIKE proteins (for example, DCL1) in plants [18]. The mature miRNAs are incorporated into Agonaute complexes that target degradation or translational repression of mRNAs [12]. As a result, miRNAs play important roles in plant development, including cell patterning and organ development, hormone signaling, and response to environmental stresses such as cold, heat, pathogens and salinity.

Mature miRNAs are often identified by computational analysis and/or experimental approaches such as cloning and sequencing [19-22]. As of March 2009, release 13.0 of the miRBase database contains 3,788 plant miRNA entries [23]. Although many transcription and phytohormonal factors are the targets of miRNAs and are predicted to play a role in cotton fiber development, the small RNA data are limited in cotton partly because cotton genome sequence is unavailable [24]. Only a dozen miRNAs have been identified through computational analysis of cotton ESTs [25] and low-throughput sequencing [26]. Few precursor structures are deposited in the miRBase [27]. A recent study using high-throughput sequencing found 34 conserved miRNAs and eight EST loci encoding conserved miRNAs in cotton [28]. To enrich our knowledge of small RNAs in cotton fiber development, we analyzed miRNAs during early stages of fiber and ovule development. We sequenced and analyzed approximately 4 million small RNAs in cotton leaves, immature ovules, and fiber-bearing ovules. The 24-nucleotide small RNAs were highly enriched in fiber-bearing ovules in cotton. We found 27 conserved families of miRNAs, identified 4 new miRNAs, and predicted 32 miRNA precursors representing 19 unique families. A total of 223 miRNA targets were computationally predicted, and a subset of these was experimentally validated. Many miRNAs, including novel miRNAs, were repressed during early stages of fiber development, which was consistent with upregulation of eight targets tested. An enrichment of siRNAs in fiber-bearing ovules and down-regulation of miRNAs in fibers suggest important roles for small RNA-mediated gene regulation in the process of rapid fiber cell development.

## Results

### Distribution of small RNAs in cotton

**Figure 1:**
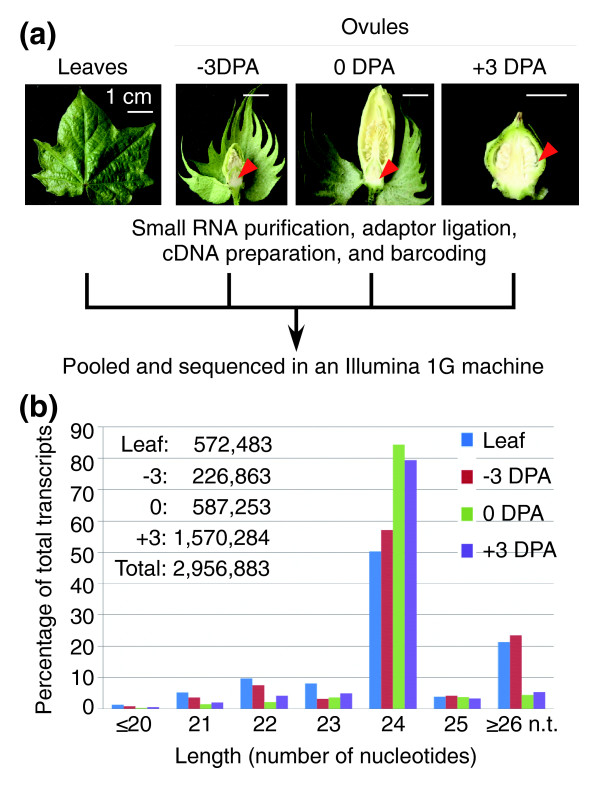
Sequencing flow chart and size distribution of small RNAs in cotton. **(a) **Flow chat of small RNA library construction and sequencing. The plant materials included seedling leaves, dissected ovules 3 days prior to anthesis (-3 DPA), on the day of anthesis (0 DPA), and 3 days post-anthesis (+3 DPA). Red arrows indicate the location of materials harvested for RNA extraction. **(b) **Size distribution of small RNAs in leaves and ovules at -3 DPA, 0 DPA, and +3 DPA. High-level accumulation of 24-nucleotide small RNAs in the ovules (0 and +3 DPA) may result from overproduction of siRNAs during early stages of ovule and fiber development. Inset: total small RNA reads after removal of other cellular RNA sequences.

To characterize small RNAs in cotton, we made four barcoded sequencing libraries using total RNAs extracted from leaves, immature ovules (3 days prior to anthesis, -3 DPA), ovules with fiber cell initials (on the day of anthesis, 0 DPA), and young fiber-bearing ovules (3 days post-anthesis, +3 DPA) in *Gossypium hirsutum *L. cv. Texas Marker-1 (TM-1) (Figure 1a). A total of 4,104,491 sequence reads of 17 to 32 nucleotides in size were generated in a pooled sample containing four barcoded libraries using an Illumina 1G Genome Analyzer. The reads were parsed into each library using a barcode base at the 5' end and an adaptor base at the 3' end. After removal of adaptor sequences, we identified the reads matching known cellular small RNAs, mitochondrial, and plastid sequences (approximately 6%). A large amount of raw reads, ranging from 6.4% in the ovules to 53.8% in the leaves, matched rRNAs (Table 1). This suggests that a high proportion of rRNAs are degraded in leaves. Alternatively, the rRNA genes in leaves may be subjected to silencing or nucleolar dominance via RNA-mediated pathways [29].

**Table 1:** Statistics of small RNA sequence reads

	All reads (%)					Distinct reads (%)				
		
Library	Leaf	-3 DPA	0 DPA	+3 DPA	Total	Leaf	-3 DPA	0 DPA	+3 DPA	Total
Matching CGI9 (EST assembly)	51.60	31.83	9.48	10.58	25.92	13.91	12.12	4.52	3.97	5.54
Matching *G. raimondii *(genomic trace reads)	61.25	42.67	24.99	24.57	38.42	26.83	23.47	19.26	17.56	18.66
Matching miRNAs	0.80	1.82	0.82	1.19	1.06	0.09	0.13	0.05	0.06	0.05
Other cellular RNAs	57.88	39.10	8.21	9.39	27.96	17.87	14.92	3.55	3.03	5.95
rRNA	53.83	29.56	6.37	7.03	24.47	16.25	11.42	2.82	2.20	4.88
tRNA	3.09	7.97	1.48	2.00	2.82	0.90	2.54	0.45	0.58	0.68
snoRNA	0.02	0.31	0.01	0.02	0.05	0.03	0.14	0.01	0.02	0.02
snRNA	0.02	0.04	0.01	0.01	0.01	0.03	0.06	0.02	0.01	0.02
Mitochondria	0.02	0.17	0.04	0.04	0.04	0.05	0.27	0.05	0.04	0.06
Chloroplast	0.91	1.05	0.29	0.30	0.57	0.62	0.49	0.20	0.18	0.29
Total raw reads	1,359,250	372,521	639,801	1,732,919	4,104,491	526,304	191,591	505,504	1,180,742	2,169,534

snoRNA, small nucleolar RNA; snRNA, small nuclear RNA.

A total of 2,956,883 sequence reads were grouped into 2,169,534 distinct reads, some of which partially overlapped (Figure 1b and Table 1). These sequences were analyzed using BLAST against the cotton EST assembly the Cotton Gene Index (CGI) version 9, which contains 350,000 ESTs [3]. Only 2.1 to 3.3% (average of approximately 2.3% or 49,899) of the distinct small RNA reads in leaves, immature ovules, and fiber-bearing ovules matched available cotton ESTs in the databases. Among them, 4,497 21-nucleotide small RNAs perfectly matched 3,203 ESTs, many of which were known miRNAs (see below), whereas 10,676 24-nucleotide small RNAs matched 12,036 ESTs. Approximately 500-Mb (approximately 0.6× genome equivalent) of *G. raimondii *whole-genome shotgun (WGS) trace reads were produced by the Department of Energy Joint Genome Institute [30] in a community sequencing project (Proposer: Andrew Paterson). *G. raimondii *is one of the probable progenitors for the allotetraploid cotton *G. hirsutum*. Over 15% of small RNA sequences in four libraries matched the WGS trace reads. Of these, 5,597 21-nucleotide small RNAs matched 13,872 WGS trace reads, most of which were known miRNAs, whereas 52,630 24-nucleotide small RNAs were mapped onto 233,999 WGS trace reads. Although these WGS trace reads represent only a small fraction of the *G. raimondii *genome, a nearly five-fold increase of the matches between 24-nucleotide small RNAs and WGS trace reads compared to the ESTs suggests that approximately 80% of the 24-nucleotide small RNAs sequenced are produced in intergenic regions, repeats, and transposons as they lack corresponding sequences in the large collection of cotton ESTs. Moreover, >85% (1,846,442) of the distinct sequences were singletons, which are reminiscent of the high number of singletons observed in *Arabidopsis *[21]. The data suggest that a quarter million to a million small RNA sequences in each tissue are far from saturation of the small RNA repertoire in cotton.

### The most abundant small RNAs in cotton ovules are 24 nucleotides long

The most abundant size of cotton small RNAs is 24 nucleotides, followed by 26 nucleotides or longer and 22 nucleotides (Figure 1b). Interestingly, 78 to 84% of small RNAs in the ovules (0 DPA) and fiber-bearing ovules (+3 DPA) were 24 nucleotides long. In *Arabidopsis*, the distribution of 24-nucleotide small RNAs is approximately 43% in leaves, approximately 61% in inflorescences, and approximately 41% in seeds [31]. The 24-nucleotide small RNAs mainly consist of siRNAs that are associated with repeats and transposons [20,32]. The high levels of 24-nucleotide small RNAs in *Arabidopsis *inflorescences and developing cotton ovules compared to those in *Arabidopsis *and cotton leaves may suggest repression of these elements in ovules or inflorescences. Alternatively, repeats and other elements are normally repressed in the leaves but activated during rapid cell development. The consequence of 24-nucleotide small RNAs on fiber development remains to be investigated after the cotton genomes are sequenced [24].

Many 24-nucleotide small RNAs were apparently derived from transposons, including 10,499 and 9,869 24-nucleotide small RNAs from copia-like and gypsy-like retrotransposons, respectively [33]. A large number (73,001) of 24-nucleotide small RNAs matched unknown repetitive sequences, suggesting that transposons and repeats are highly diverged between cotton and other plants whose genomes are sequenced. The number of 24-nucleotide small RNA reads was normalized to transcripts per quarter million (TPQ) per megabase of repetitive sequences. The data indicated that the number of small RNAs matching known repeats and transposons present in the *G. herbaceum *and *G. raimondii *genomes was similar, but the number matching specific repetitive sequences, mostly the retrotransposons and transposons, was higher in *G. raimondii *than in *G. herbaceum *(Additional data file 1). Although the available repetitive sequences are relatively small in cotton, a relatively high amount of 24-nucleotide small RNAs may suggest repression of repetitive sequences, including retrotransposons of *G. raimondii *origin in tetraploid cotton.

### Identification of miRNAs in cotton

We adopted the common criteria [34] to identify known miRNAs and/or precursors in cotton. First, a cotton miRNA must have sequence conservation and homology to orthologous miRNAs in other species. Second, if a miRNA matches known ESTs, the stem-loop structure clearly shows miRNA and miRNA* in the opposite arm of a duplex. Many miRNAs contain a sequenced miRNA* species with 2-nucleotide 3' overhangs, providing strong evidence for a DCL1-processed stem-loop. Third, base paring occurs extensively within the region of the miRNA and an arm of a predicted hairpin. Finally, the miRNA contains minimal asymmetric bulges (less than four).

Using these criteria, we identified 27 miRNA families that were present in one or more tissues examined in *G. hirsutum *L. (Table 2). Ten of them (Gh-miR156, 159, 164, 165/166, 167, 168, 171, 172, 535, and 894) were present in all four tissues. The majority of miRNAs were detected in leaves. Gh-miR390 and 393 were found in the fiber-bearing ovules (0 and +3 DPA) but were absent in immature ovules (-3 DPA). Several miRNAs that were recently identified by deep-sequencing in *Arabidopsis*, including miR827 and miR828 [21], were also found in cotton. Gh-miR165/166 and a candidate novel miRNA (see below) were most abundant, followed by Gh-miR167, 168, 156/157, 172, 171, 390, 535, and 894. The abundance of Gh-miR165/166 was 3,979 TPQ in leaves and 7,340, 1,902, and 2,728 TPQ in ovules at -3, 0, and +3 DPA, respectively. TPQ varied from one tissue to another, suggesting differential accumulation of miRNAs during leaf, ovule and fiber development.

**Table 2:** MicroRNAs detected by sequencing and their target gene families predicted in cotton

Sequence (5'-3')*	Gh-miRNA	Total^†^	Leaf	-3 DPA	0 DPA	+3 DPA	Number of targets	Target gene family description
UUGACAGAAGAUAGAGAGCAC	156/157	36.6	65.9	19.8	8.9	38.6	20	Squamosa promoter-binding factors, Ser/Thr protein phosphatase
UUUGGAUUGAAGGGAGCUCUA	159	11	17.5	20.9	7.2	8.6	4	Beta-ketoacyl-CoA synthase
UGCCUGGCUCCCUGUAUGCCA	160	5.5	27.1	0	1.3	0	5	Auxin response factor (ARF) family
UCGAUAAACCUCUGCAUCCAG	162	0.6	2.2	0	0	0.3	4	Allyl alcohol dehydrogenase
UGGAGAAGCAGGGCACGUGCA	164	7.9	22.7	1.1	3.4	5.1	2	NAC domain transcription factors
UCGGACCAGGCUUCAUUCCCC	165/166	3,250.5	4,058.6	7,506.8	1,928.1	2,835.4	10	Class III HD-Zip proteins
UGAAGCUGCCAGCAUGAUCUCA	167	232.9	107	22	176.2	330.5	7	Auxin response factor (ARF) family, glycoprotease
UGCUUGGUGCAGAUCGGGAC	168	144.9	72.9	5.5	7.7	242.6	4	Argonaute 1, F-box proteins
CAGCCAAGGAUGACUUGCCGG	169	0.8	4.4	0	0	0	11	Heme activating protein (HAP2), CCAAT-binding transcription factors
UGAUUGAGCCGUGCCAAUAUC	170/171	26.7	132.3	3.3	0.4	1.4	8	Hairy meristem/Scarecrow-like 6 transcription factors
AGAAUCUUGAUGAUGCUGCAU	172	51.3	193.9	15.4	8.5	20.5	21	APETALA2, AHAP2-like factors, Target of EAT1 (TOE1)
UGGACUGAAGGGAGCUCCCUC	319	0.1	0.4	0	0	0	7	TCP family transcription factors
AAGCUCAGGAGGGAUAGCGCC	390	8.1	15.3	0	11.1	5.6	11	TAS3, leucine-rich repeat transmembrane protein kinase
UCCAAAGGGAUCGCAUUGAUUU	393	3.1	0.9	0	13.2	0.6	11	Transport inhibitor response 1 (TIR-1)
UUCCACAGCUUUCUUGAACUG	396	1.4	6.1	0	0.9	0.2	31	ATCHR12 transcriptional regulator, growth regulating factors (GRF)
UCAUUGAGUGCAGCGUUGAUG	397	0.1	0.4	0	0	0	13	Laccase/copper ion binding proteins, diphenol oxidase
UGCCAAAGGAGAUUUGCCCGG	399	1.1	4.8	0	0	0.3	2	MYB family transcription factor, TIR-1
AUGCACUGCCUCUUCCCUGGC	408	0.1	0.4	0	0	0	10	Blue copper proteins, uclacyanin 3
UGUGGGAGAGUUGGGCAAGAAU	2948	3.4	10	0	1.7	2.1	5	Sucrose synthase, glucose-methanol-choline (GMC) oxidoreductase
UCUUGCCUACUCCACCCAUGCC	472/482	6.2	31.4	0.0	0.0	0.2	9	NBS-type resistance protein
UGCAUUUGCACCUGCACCUUC	530	1.9	9.6	0	0	0.2	5	C2H2 transcription factors, bHLH family protein
UGACAACGAGAGAGAGCACGU	535	11.9	45.9	1.1	1.3	5.1	4	Squamosa promoter-binding factors
UUAGAUGACCAUCAACAAACA	827	0.3	1.7	0	0	0	1	Unknown
UCUUGCUCAAAUGAGUAUUCUA	828	0.1	0.4	0	0	0	7	MYB family transcription factors
GUUUCACGUCGGGUUCACCA	894	26.8	38.9	55.1	32.4	16.2	4	Responsive to dessication 20
UAUACCGUGCCCAUGACUGUAG	2947	11.2	46.3	0	1.7	3.7	1	Serine/threonine protein phosphatase
ACUUUUGAACUGGAUUUGCCGA	2949	5.7	8.3	0	6.8	5.1	6	Endosomal protein
UGGUGUGCAGGGGGUGGAAUA	2950	0.8	3.9	0	0	0.2	5	Gibberellin 3-hydroxylase/anthocyanidin synthase
UUGGACAGAGUAAUCACGGUCG	GhmiRcand1	3,683.5	5,684	14,594.7	311.6	2,638.9	3	NAC domain transcription factors

*Most abundant variant shown. Gh: *Gossypium hirsutum*; The miR2947 precursor was derived from a *G. arboreum *EST. ^†^Abundance is normalized to transcripts per quarter million (TPQ) and rounded to nearest tenth.

### Conservation of miRNAs in cotton and other species

We compared cotton small RNAs (mainly 21-nucleotide small RNAs) with the miRNAs identified in moss (Pp, *Physcomitrella patens*), the eudicots thale-cress (At, *Arabidopsis thaliana*), grape, and black cottonwood (Pt, *Populus trichocarpa *Torr. & Gray), and the monocots rice (Os, *Oryza sativa *L.), sorghum (Sb, *Sorghum bicolor *L.), and maize (Zm, *Zea mays *L.) (Table 3), whose genomes were partially or completely sequenced. Among 27 miRNA families analyzed, 9 (Gh-miR156/157, 160, 165/166, 167, 170/171, 319, 390, 408, and 535) were conserved among moss, eudicots and monocots, and 23 existed in both monocots and eudicots but not moss. Three miRNA families (miR472/482/1448, 479, and 828) were found in eudicots but not in monocots. These data suggest that many miRNAs are conserved among plant species.

**Table 3:** Conservation of miRNAs in cotton and other plants

	Embryophyte	Eudicots				Monocots		
			
miRNA	Moss	Thale-cress	Cottonwood	Grape	Cotton	Rice	Sorghum	Maize
156/157	3	12	11	9	H,P,M,S	12	5	11
159		3	6	3	H,N,M,T	6	2	4
160	9	3	8	6	H,M,T	6	5	6
162		2	3	1	H,P,M,S	2		1
164		3	6	4	H,N,P,M,T	6	3	4
165/166	13	9	17	8	H,N,P,M,T,S	14	7	13
167	1	4	8	5	H,N,P,M,T	10	7	9
168		2	2	1	H,N,M,T	2	1	2
169		14	32	25	H	17	9	11
170/171	2	4	14	9	H,P,M	9	6	11
172		5	9	4	H,N,P,M,T,S	4	5	5
319	5	3	9	5	H	2	1	4
390	3	2	4	1	H,P,M,T	1		
393		2	4	2	H,P,M	2	1	1
394		2	2	3	P	1	2	2
396		2	7	4	H,P	6	3	4
397		2	3	2	H,M	2		
398		3	3	3	P,M	2		
399		6	12	9	H,P	11	9	6
408	2	1	1	1	H,M	1		1
479			1	1	P			
472/482		1	4	1	H,P,S			
530			2		H	1		
535	4			5	H,M	1		
827		1	1		H,P,S	3		
828		1		2	H,T			
894	1				H			

H, homology; P, precursors detected; M, microarrays; N, Northern validated; T, targets identified; S, miRNA* sequenced. Numbers represent the number of precursor loci deposited in miRBase 13.0. Moss, *Physcomitrella patens *(Hedw.) Bruch & Schimp. in B. S. G.; thale-cress, *Arabidopsis thaliana *(L.) Heynh.; grape, *Vitis vinifera *L.; black cottonwood, *Populus trichocarpa *(Torr. & A. Gray) Brayshaw; cotton, *Gossypium hirsutum *L.; rice, *Oryza sativa *L.; sorghum, *Sorghum bicolor *(L.) Moench; and maize, *Zea mays *L.

### Stem-loop structures of miRNAs and identification of novel miRNAs in tetraploid cotton

Hairpin stem-loop structures were visualized using the sir graph tool in the UNAFold package [35]. Thirty-two miRNA precursors including 19 unique families were identified in CGI9 using MIRcheck [19,36] (Additional data file 2), which represented only a small portion of the miRNA families (Table 3) identified in this study. This suggests that miRNA precursors have been underrepresented in the EST database, despite a large number of EST sequencing efforts in cotton. The ESTs are primarily derived from the allotetraploid cotton (*G. hirsutum*) and close relatives of its probable progenitors, *Gossypium arboreum *and *Gossypium raimondii*. Many ESTs are partial sequences of full-length cDNAs, and the representation of ESTs in early stages of fiber development is relatively low [3].

**Figure 2:**
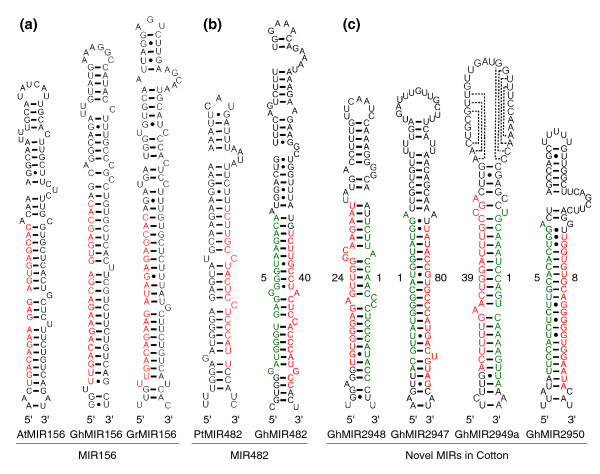
Stem loop structures of core pre-miRNAs. **(a) **Stem-loop structures of miR156 in *A. thaliana *(*AtMIR156*), *G. hirsutum *(*GhMIR156*), and *G. raimondii *(*GrMIR156*) showing overall conserved structures among them and slightly different sequence composition and structure between *GhMIR156 *and *GrMIR15*6. **(b) **The conserved miR482 is located in the 3' end of the stem in *Populus trichocarpa *(*PtMIR482*) and *G. hirsutum *(*GhMIR482*). **(c) **Stem-loop structures of four novel miRNAs (Gh-MIR2948, Gh-MIR2947, GhMIR2949a, and GhMIR2950) in *G. hirsutum*. One of the three predicted pre-*GhMIR2949a *EST stem-loops is shown. Gh-miR482-5p is located in the 5' end of the stem in the new miRNA *Gh-MIR2948*. Gh-miR2948 was predicted to possess different targets from Gh-miR482 (b). Mature miRNAs and miRNA* are shown in red and green, respectively. The numbers in (b, c) indicate total miRNA and miRNA* sequence reads, respectively, in four tissues examined.

We compared the stem loop structures of a few miRNAs that contain predicted miRNA precursor hairpins in cotton with the corresponding ones in *Arabidopsis *and cottonwood (*Populus*). The stem loop structures of *AtMIR156 *and *GhMIR156 *shared many common features, including 5' UU and 3' C bulged bases adjacent to the hairpin loop region (Figure 2a). These conserved structural features have been suggested to guide the DCL1-mediated processing of miRNA precursors [19]. *GrMIR156 *was found to have a few different features such as a bulged G in the miRNA*, suggesting that *GhMIR156 *matches an EST derived from the *G. arboreum*-like subgenome in *G. hirsutum*.

The miR472/482/1448 family was recently identified in *Arabidopsis *and cottonwood [21]. miR482 in cotton was identified in the 3' end of three ESTs (TC106817, DR457519, and DT527030) and had very similar canonical miRNA sequences (Figure 2b). These ESTs may be derived from paralogous and/or homoeologous sequences in allotetraploid cotton, and their miRNAs should belong to the same family. The mature miRNA ratio of Gh-miR482 to Gh-miR482* was 8:1 (40:5 total reads). Interestingly, another miRNA, Gh-miR482-5p, was derived from the 5' end of the EST (DW517596) with 24 reads, and Gh-miR482-5* was derived from the 3' end of the EST with only 1 read, resulting in a ratio of 24:1 (miRNA to miRNA*) (Figure 2c). In the canonical miRNA sequences, the level of divergence is higher between Gh-miR482-5p and Gh-miR482 than between Gh-miR482-5p* and Gh-miR482*. Gh-miR482-5p and Gh-miR482 were in the opposite strands of different ESTs and expected to target different sets of genes. Common features such as a 4- to 5-bp bulge in the 3' end proximal to the loop were found in the stem loop structures of *GhMIR482 *and *PtMIR482*, but not in *GhMIR482-5p*. Although miRNAs can be found in both 3' and 5' ends of precursors [22], Gh-miR482-5p has not been identified in *Arabidopsis *or cottonwood and is considered a new miRNA, named Gh-miR2948. In addition, miR2948 has a miR2948* that closely matches miR482, indicating that miR2948 has likely evolved from the miR484 family. One of the Gh-miR2948 targets is predicted to encode a sucrose synthase-like gene (ES815756; Additional data file 3). Sequencing reads indicated lower levels of Gh-miR2948 in both immature and fiber-bearing ovules than in leaves (Table 2), which correlates with upregulation of the sucrose synthase gene (U73588) in early stages of fiber cell development [37].

In addition to the new miRNA Gh-miR2948, we identified three novel miRNAs and one candidate-novel miRNA in cotton using the commonly adopted criteria [34]. After extensive computational analysis of potential precursors against ESTs in CGI9, we selected the list of candidates with miRNA* sequence present on the opposite strands of predicted hairpin structures. According to miRBase, the three new cotton miRNA families were named Gh-miR2947, Gh-miR2949, and Gh-miR2950, and their corresponding loci were named *Ga-MIR2947*, *Gh-MIR2949*, and *Gh-MIR2950*, respectively (Figure 2c). The precursor of Gh-miR2947 was derived from an EST of *G. arboreum*, and the corresponding locus was named *Ga-MIR2947*. The miRNA to miRNA* ratios were 80:1 in Gh-miR2947, 39:1 in Gh-miR2949, and 8:5 in Gh-miR2950. Gh-miR2949a, b, and c matched three ESTs (AI054573, EV497941, TC94314, respectively) that are putative precursors (Additional data file 2), implying multiple members of this miRNA family. Gh-miR2947 was predicted to target a cotton EST encoding a putative serine/threonine protein phosphatase 7 homolog. Six ESTs encoding endosomal proteins were the predicted targets of Gh-miR2949 (Additional data file 3). Among five putative EST targets of Gh-miR2950, two encode putative gibberellin 3-hydroxylase 1 in *G. hirsutum*. The candidate-novel miRNA (Gh-miRcand1) is 22 nucleotides long and has a canonical 5' U. Gh-miRcand1 had the most abundant sequence reads (approximately 39,860; Table 2) and was detected by small RNA blot analysis. Moreover, it matched three EST targets that encode NAC domain transcription factors, but its potential precursors were not found in the EST databases.

### Differential expression of conserved miRNAs in cotton

**Figure 3:**
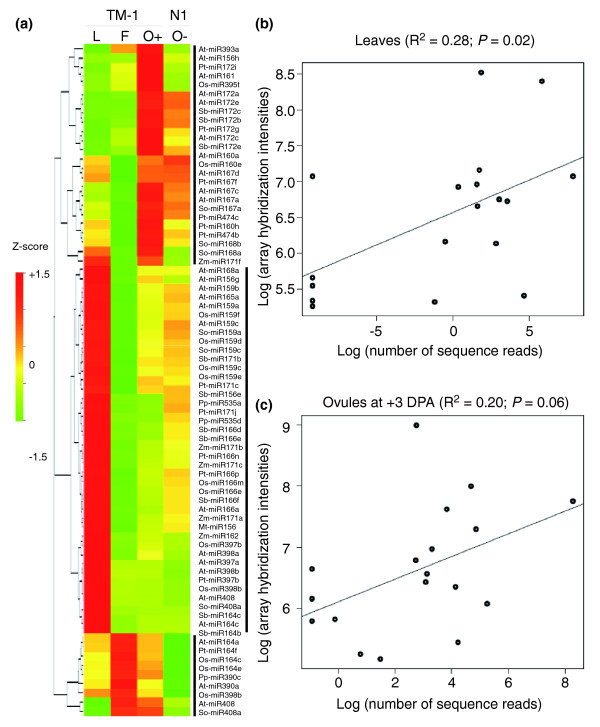
Differential accumulation of miRNAs in microarray and sequence assays. **(a) **Hierarchical cluster analysis of miRNA expression variation in leaves (L), fibers (F; +7 DPA) and fiber-bearing ovules (O+; +3 DPA) of TM-1 and ovules without fibers (O-; +3 DPA) of the *N1N1 *mutant (N1). At, *Arabidopsis thaliana*; Gm, *Glycine max*; Mt, *Medicago truncatula*; Os, *Oryza sativa*; Pp, *Physcomitrella patens*; Pt, *Populus trichocarpa*; Sb, *Sorghum bicolor*; So, *Saccharum officinarum*; Zm, *Zea mays*. Vertical lines indicate similar expression patterns of miRNAs in 'blocks'. **(b) **Positive correlation of miRNAs between sequencing frequencies and microarray hybridization intensities detected in cotton leaves (R^2 ^= 0.28; *P *= 0.02; degrees of freedom (df) = 16). **(c) **Positive correlation of miRNAs between sequencing frequencies and microarray hybridization intensities detected in cotton ovules (+ 3 DPA; R^2 ^= 0.20; *P *= 0.06; df = 16).

To further characterize cotton miRNAs, we employed miRNA microarrays (CombiMatrix, version 9.0) [38] to determine miRNA accumulation patterns in cotton fibers and non-fiber tissues. Each microarray interrogated 85 distinct miRNAs and 23 tasiRNAs in *Arabidopsis *(At), 62 miRNAs in black cottonwood (Pt), 10 in barrel medic (Mt, *Medicago truncatula *Gaertn), 1 in soybean (Gm, *Glycine max *L.), 73 in rice (Os), 8 in maize (Zm), 19 in sorghum (Sb), 8 in sugarcane (So, *Saccharum officinarum *L.), and 26 in moss (Pp). A total of 111 miRNAs comprising 27 families derived from these species were expressed above the detection level (Additional data file 4) in cotton. Microarray results confirmed the expression of 21 conserved miRNAs present in the sequencing libraries (Table 2) and revealed an additional 55 miRNAs that were expressed in one or more tissues, including leaves (L), fibers (F; +7 DPA), and fiber-bearing ovules (O+; + 3 DPA) of *G. hirsutum *L. cv. TM-1 and ovules without fibers (O-; +3 DPA) of the *N1N1 *naked seed mutant with reduced fiber production in TM-1 background (Figure 3a). Although cross-species hybridization based assays may introduce false positives, additional miRNAs detected in microarrays suggest that the pool of miRNAs identified in this study is unsaturated by sequencing.

The expression patterns of miRNAs were clustered into three blocks (Figure 3a; Additional data file 4). First, 44 (out of 76 or approximately 58%) miRNAs belonging to 12 families (miR168, 171, 156, 159, 165, 535, 166, 162, 397, 398, 408, and 164) were expressed at higher levels in leaves than in ovules and fibers. Second, nine (approximately 12%) miRNAs (At-miR164a, Pt-miR164f, Os-miR164c, Os-miR164, Pp-miR390c, At-miR390a, Os-miR398b, At-miR408, and So-miR408a) belonging to four families (miR164, 390, 398, and 408) were expressed at higher levels in fibers (+7 DPA) than in fiber-bearing ovules in TM-1 but at very low levels in leaves (TM-1) and ovules without fibers (*N1N1 *mutant). Finally, 25 miRNAs of 10 families (miR393, 156, 172, 161, 395, 160, 167, 474, 168, and 171) were highly expressed in the ovules with and without fibers. Seven miRNAs (At-miR393a, miR156h, miR161, Pt-miR172i, Os-miR395t, So-miR168a, and Zm-miR171f) accumulated at higher levels in the ovules with fibers than in the ovules without fibers (*N1N1 *mutant). Note that the hybridization intensities of some miRNAs in different species may not be directly related to that of corresponding cotton miRNAs because of potential sequence variation between cotton and other plant miRNAs.

**Figure 4:**
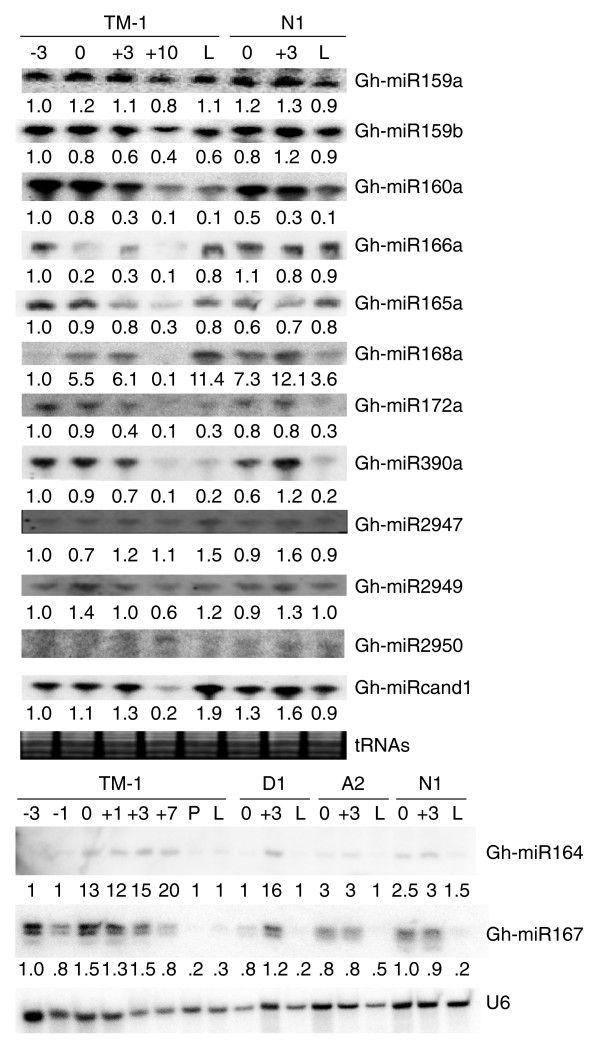
Small RNA blot analysis of miRNA accumulation in cotton leaves, fiber-bearing ovules, and fibers (n = 2). U6 or tRNAs were used as hybridization and RNA loading controls. Gh-miRNAs are shown on the right. TM-1, *G. hirsutum *cv. TM-1; D1, *G. thurberi*; A2, *G. arboreum*; N1, *N1N1 *lintless mutant of TM-1; -1 and -3, 1 and 3 days prior to anthesis, respectively; 0, on the day of anthesis; +1, +3, and +5, 1, 3, and 5 days post-anthesis (DPA), respectively; +7 and +10, fibers harvested at 7 and 10 DPA, respectively; L, leaves; P, petals. Note that doublets in miR167 were probably produced from precursors of multiple miRNA loci in cotton. The levels of Gh-miR2950 were very low and not quantified. A fragment present in fiber in the Gh-miR2950 blot was larger than 21 nucleotides and probably an artifact.

Among 16 miRNA families examined, the relative expression levels estimated from microarrays and sequencing results were related in TM-1 leaves (R^2 ^= 0.29, *P *= 0.02; Figure 3b) and in fiber-bearing ovules (+3 DPA, R^2 ^= 0.20, *P *= 0.06; Figure 3c). A marginal significance level may indicate variability in RNA preparations used in the two independent experiments. These values also correlated with the data obtained by small RNA blots (Figure 4). The microarray and blot data correlated more strongly with each other than with sequencing frequencies probably because the sensitivity and/or variability was high in sequencing [21].

### Accumulation of miRNAs during ovule and fiber development

Using small RNA blot analysis, we examined and validated the expression patterns of nine miRNAs during ovule and fiber development in TM-1 and *N1N1*. Many miRNAs tested, including Gh-miR159, 160, 165/166, 168, 172 and 390, accumulated at low levels in fibers (+7 DPA) and fiber-bearing ovules (+3 DPA) relative to leaves and immature ovules (-3 and 0 DPA) (Figure 4). Gh-miR167 and Gh-miR164 accumulated at higher levels in fiber-bearing ovules than in other tissues examined. Gh-miR167 detected doublets, which probably resulted from processing of several miRNA precursors in different loci or in different progenitors. To test this, we included putative diploid progenitors (A2 and D1 species) in the small RNA blots. Both fragments were present in each diploid progenitor, ruling out a possibility of different miR167 species in the diploid cotton tested.

Compared to miR159 and miR160, the expression levels of novel miRNAs (Gh-miR2947 and Gh-miR2949) were relatively low (Figure 4). Both Gh-miR2947 and Gh-miR2949 accumulated at lower levels in fibers than in leaves and ovules, while Gh-miR2949 was highly expressed in the fiber-bearing ovules (+3 DPA). Gh-miRn3 was nearly undetectable but present in the ovules of *N1N1*.

miR390 accumulated at lower levels in fibers and leaves than in ovules. miR390 targets TAS3 and produces tasiRNAs that in turn regulate the expression of *ARF3 *and *ARF4*, which are responsible for auxin (Aux)/indole acetic acid (IAA) signaling, developmental timing and patterning in *Arabidopsis *[39,40]. A low level of miR390 may lead to a high level of *ARF3 *and *ARF4 *and Aux/IAA signaling during fiber elongation and leaf expansion. Interestingly, the expression levels of miR159b, 160a, 165a and 166a decreased in the ovules from -3 DPA to +3 DPA and in fibers (+10 DPA) in TM-1, but their expression levels remained relatively unchanged in the ovules at 0 and +3 DPA in the *N1N1 *mutant (Figure 4). A low level of miR390 was detected in the fibers at +10 DPA in small RNA blots, whereas miR390 accumulated at a high level in the fibers at +7 DPA using the microarrays. This may suggest miRNA expression differences or technical variation between the small RNA blot and microarray detection methods.

In contrast to many miRNAs, miR164 accumulated at higher levels in fibers and fiber-bearing ovules than in leaves and immature ovules. The microarray results indicated cotton transcripts hybridizing to the probes for the miR164 family, including At-miR164c and At-miR164a, Os-miR164b and OS-miR164e, Sb-miR164b, and Pt-miR164f, accumulated to higher levels in fibers than in wild-type leaves and ovules but not in the ovules of the naked seed mutant (Figure 3a). To compare this expression difference in the extant diploid progenitor species, we extended our expression analysis to include *G. arboreum *(A2A2) and *G. thurberi *(D1D1). In the tetraploid *G. hirsutum*, miR164 started to accumulate in the ovules at 0 and +3 DPA, and its levels increased in the fibers at 7 DPA. In the extant diploid progenitor *G. arboretum*, which also produces fibers, miR164 was barely detectable in the ovules at 0 and +3 DPA. miR164 was expressed in the ovules at 3 DPA in *G. thurberi *and at 0 and +3 DPA in the *N1N1 *mutant, both of which produced few fibers. Although the exact diploid progenitors of *G. hirsutum *L. are unknown, the data suggest that miR164 expression changed in the allotetraploids relative to the extant diploid progenitors.

### miRNA-guided target degradation in cotton

miRNAs post-transcriptionally regulate gene expression by directly cleaving mRNA [41] or inhibiting mRNA translation [42,43]. To test whether miRNAs trigger target cleavage in cotton, we predicted miRNA targets using Perl scripts and the target prediction criteria as previously described [44]. The miRNA targets were generally found in the antisense hits with three or fewer mismatches outside of the canonical miRNA sequences. A total of 233 potential targets were identified for 31 putative miRNA families, including 27 conserved and 4 novel families (Table 2; Additional data file 3). Many of the predicted targets were derived from ESTs in the early stages of fiber development [3]. Compared to *Arabidopsis*, the number of predicted targets per miRNA in cotton is large, suggesting additional paralogous and homoeologous genes in this allotetraploid species.

**Figure 5:**
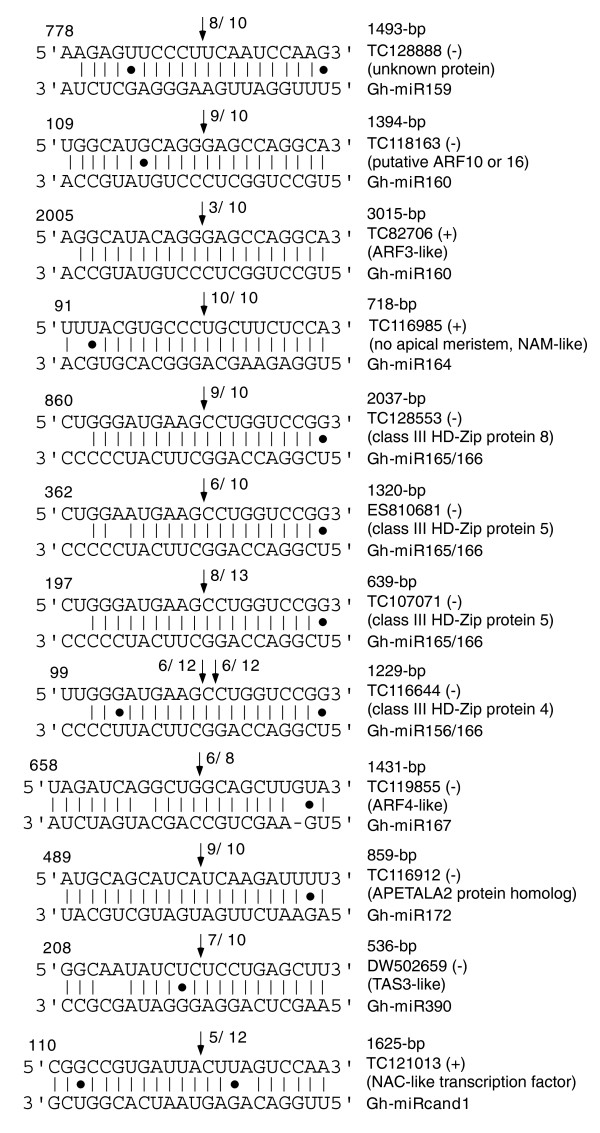
Mapping of mRNA cleavage sites by cotton miRNAs using RNA ligase-mediated rapid amplification of 5' complementary DNA ends (RLM 5' RACE). The arrows indicate the 5' ends of miRNA-guided cleavage products, and the numbers indicate the ratios of cleaved products of the total fragments that were sequenced. Only a single cleavage product was observed within the miRNA-complementary region for each examined EST target, except for a miR166 target, in which two products resulting from adjacent sites were detected. The EST targets (from 5' to 3') with accession numbers are shown in the top strand ('+', sense strand orientation; '-', antisense strand), and Gh-miRNAs are shown in the bottom strand with the orientation from 3' (left) to 5' (right). The total lengths of the target ESTs are shown above the EST accession numbers. Wobble U-G pairs are indicated by black dots. Note that Gh-miR166 had two adjacent cleavage sites in the predicted target. ARF, auxin responsive factor.

The expression patterns of putative target genes, including auxin response factors, may vary during fiber development [3,7]. To maximize the detection efficiency of miRNA-triggered target cleavage, we used an equal mixture of mRNAs from ovules and fibers in this assay. The mixed RNA may obscure the target cleavage in a specific tissue but would allow us to determine if a specific target is cleaved by a corresponding miRNA during ovule and fiber development. miRNA-guided target cleavage was examined for 12 targets of 7 miRNAs (Gh-miR159, 160, 164, 165/166, 167, 172, and 390) and one candidate-novel miRNA (Gh-miRcand1) in cotton (Figure 5). Cleavage was detected in the predicted target (encoding a NAC transcription factor) of Gh-miRcand1, and all target products of the miRNAs examined were cleaved at the predicted sites as shown in *Arabidopsis *(Figure 5, Additional data file 3). The cleaved products were usually terminated at a position corresponding to the tenth position of complementarity from the miRNA 5' end. This is characteristic of a RNA-induced silencing complex (RISC)-like processing event and provides evidence for miRNA-guided target cleavage in cotton. Within a given target, over 60% of miRNA-triggered degradation products mapped on the predicted sites, except for miR160 and Gh-miRcand1.

Gh-miRcand1 had most abundant reads, but its precursor was not identified. Gh-miRcand1 triggered degradation for a predicted target that encodes a putative NAC transcription factor at a relatively low frequency, suggesting alternative targets or changes in target specificity in cotton. *Arabidopsis *miR390 cleaves *TAS3 *transcripts, which in turn trigger degradation of *ARF3 *and *ARF4*, which affect lateral organ development and the transition from juvenile to adult stages [39,40]. Similarly, Gh-miR390-guided *TAS3 *activity in cotton suggests a role for miR390 and *TAS3 *in the developmental transition from protodermal cells in the ovules to fiber cell initiation and elongation. miR160 is predicted to target an *ARF3*-like EST (TC82706) in cotton, but it triggered cleavage of the putative target at a very low frequency (Figure 5). Instead, miR160 triggered degradation in the predicted site for approximately 90% of the products of another target (TC118163) that encodes a putative ARF10 or ARF16 (Additional data file 3). This may suggest divergence between miRNA and target specificity in cotton as observed for miR159 and miR319 targets in *Arabidopsis *[45]. Several predicted target mRNAs encoding putative ARF4 (also targeted by TAS3 in *Arabidopsis*) and ARF8 were cleaved by miR167. miR164 triggered degradation of a no apical meristem (NAM)-like target in cotton, and miR166 guided cleavage of three targets encoding class III HD-ZIP proteins 5 and 8.

### Expression correlation between miRNAs and their targets

**Figure 6:**
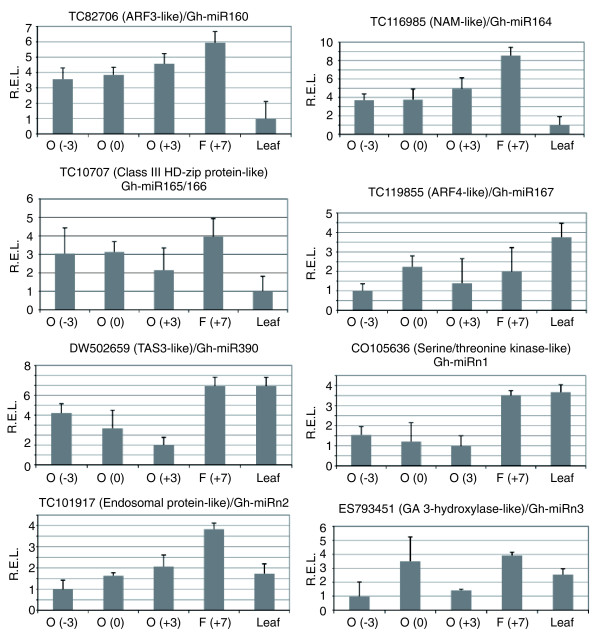
Quantitative RT-PCR analysis of predicted targets of miRNAs, including three novel miRNAs, in cotton. O (-3), O (0), O (+3), F (+7), and Leaf indicate RNA samples from the ovules at -3 DPA, 0 DPA, and +3 DPA, fiber at +7 DPA, and leaves, respectively. The label above each plot indicates the EST accession number followed by the predicted gene function and the corresponding miRNA. Relative expression levels (REL) were calculated using *HISTONE H3 *as a control.

If miRNAs degrade target mRNA transcripts, their expression levels should be negatively correlated. To test this, we compared the expression patterns of predicted miRNA targets in quantitative RT-PCR (qRT-PCR) assays with miRNA accumulation levels in small RNA blot analysis. The expression levels of some miRNA targets are inversely correlated with the accumulation levels of corresponding miRNAs. For example, TC107071 encoding a homolog of *Arabidopsis *HD-ZIP III protein was cleaved by Gh-miR165/166 (Figure 5) and expressed at relatively high levels in fibers (Figure 6), during which the Gh-miR165/166 levels were relatively low (Figure 4). The expression levels of miR390 and TAS3-like (DW502659) were also negatively correlated. TAS3-like was highly expressed in fibers and leaves (Figure 6), in which miR390 accumulated at low levels (Figure 4). Negative correlation was also observed between Gh-miR164 and its target (TC116985) encoding a NAM-like protein, between Gh-miR2947 and its target (CO105636) encoding a predicted serine/threonine kinase, and between Gh-miR2949 and its target (TC101917) encoding a putative endosomal protein. Interestingly, Gh-miR2949 accumulated at higher levels in ovules (0 DPA) than in fibers (Figure 4), and its target was expressed at lower levels in the ovules than in the fibers (Figure 6). Gh-miR2947 accumulated at lower levels in fibers than in ovules, and its target was expressed at higher levels in fibers than in ovules.

Auxin plays an important role in cotton fiber development [1,6,46]. miR160, miR167, and miR390 are involved in the auxin signaling transduction pathway through regulating the expression of *ARF6*, *ARF8*, *ARF10*, and *ARF16*. In cotton, Gh-miR167 was expressed at higher levels in fibers than in leaves (Figure 4), and the predicted target TC119855 was expressed at lower levels in fibers than in leaves (Figure 5). Moreover, DT565265, which encodes a putative ARF8, was highly expressed in immature ovules at -3 DPA, and its expression level was dramatically reduced after fiber initiation from 0 to 5 DPA [3]. Our preliminary analysis of microarray data indicated that TC118163, which encodes putative ARF10 or 16, was repressed in fiber cell initials compared to ovules at 2 DPA (data not shown). The predicted target (ES793451) of Gh-miR2950, which encodes a putative gibberellin 3 hydroxylase, were expressed at relatively high levels in the fibers and ovules (0 DPA).

The expression levels of two other targets of miR165/166, TC87226 and TC116644, which encode putative HD-ZIP III homologs, were not obviously correlated with Gh-miR166a accumulation levels (data not shown).

## Discussion

### Accumulation of 24-nucleotide small RNAs during early stages of fiber development

The high-throughput sequencing analysis revealed a burst of 24-nucleotide small RNAs during early stages of fiber and ovule development. Although the reads of other small RNAs may be inflated by relatively low abundance of miRNAs in these tissues, the total amount of miRNA reads is relatively small. The amount of 24-nucleotide small RNAs accounts for 78 to 84% of total small RNA reads (approximately 3 million reads) in fiber-bearing ovules (from 0 to +3 DPA), but only approximately 50% in leaves and approximately 57% in immature ovules. Most 24-nucleotide small RNAs are derived from endogenous repeats, including transposons and pseudogenes, and are known as repeat associated siRNAs (rasiRNAs). Although encoding loci for many rasiRNAs are unknown, a proportion of siRNAs is derived from lineage-specific retrotransposons in diploid cotton species, including *G. raimondii *and *G. herbaceum *[33]. These findings are reminiscent of uniparental inheritance of polymerase IV (PolIV)-associated 24-nucleotide siRNAs in young ovules and developing seeds [47] and rapid changes in siRNAs and miRNA expression in closely related species and allotetraploids of *Arabidopsis *[48]. Whether or not the enrichment of 24-nucleotide siRNAs in cotton ovules and fibers is biased toward one progenitor or another in the cotton allotetraploids remains to be investigated. Many siRNAs matched repeats and transposons in the WGS trace reads of *G. raimondii*, suggesting that a complete genome of cotton will help elucidate the functions of these siRNAs and other small RNAs, including miRNAs, during cotton fiber development [24].

rasiRNAs induce RNA-directed DNA methylation through a pathway that involves RNA dependent RNA polymerase 2 (RDR2), DCL3, PolIVa and PolV [49]. Effector complexes containing rasiRNAs, Argonaute (AGO)4, and PolV directed DNA methylation and chromatin modifications through the activities of additional factors, including Domains rearranged methylase 1 and 2 (DRM1 and DRM2), Chromomethylase 3 (CMT3), and SU(VAR)3-9 homologue 4 (SUVH4) [13]. The rasiRNAs are virtually absent in the single-cell alga *Chlamydomonas reinhardtii *[50] and constitute less than 10% of total reads in moss [51], suggesting an expansion of siRNAs in land plants probably due to increased amounts of repetitive DNA and transposons, and in cotton to polyploidy. Rapid cell division and expansion during early stages of fiber development may reprogram chromatin structures and lead to increased siRNA production, which in turn induces repressive chromatin structures involving DNA and histone methylation. Alternatively, the homoeologous genomes in allotetraploid cotton may be reprogrammed during fiber development, which activates rasiRNA production. Indeed, in *Arabidopsis *allotetraploids, homoeologous-specific centromeric siRNAs are associated with DNA methylation [52]. Whether or not the repeat-associated genes are methylated during fiber development remains to be investigated. In addition, the high amount of degraded rRNA fragments in leaves indicates that uniparental rRNA genes are subjected to nucleolar dominance in leaves of the allotetraploid cotton, probably through an RNA-dependent mechanism as observed in a recent study [29].

### Conserved and new miRNAs in cotton

We identified members of 27 conserved families and 4 novel miRNA families in 6 putative loci using high-throughput sequencing and computational analysis. Multiple miRNA variants were detected in cotton, which is probably associated with the polyploid nature of *G. hirsutum*. For a given miRNA-encoding locus in a diploid, there are two loci in an allotetraploid or more if the locus is duplicated in the diploid progenitor species or duplicated after the polyploidy event. Without the complete genome sequence, it is difficult to evaluate these possibilities.

Only seven miRNA precursors (miR160, 164, 827, 829, 836, 845 and 865) were previously reported by computational analysis of cotton ESTs [27]. In another study sequencing 6,691 clones from 11 ovule small RNA libraries, 2,482 candidate small RNAs were found [26]. Among them, three miRNAs (miR172, miR390 and At-miR853-like) were identified and confirmed. In this study, we identified 32 miRNA precursors, including 19 unique miRNA families in cotton. A total of 25 new miRNA precursors were found using the ESTs primarily derived from *G. hirsutum*, *G. arboreum*, and *G. raimondii *[3,24]. Among 19 conserved miRNAs with predicted precursors matching existing ESTs, 16 were detected by miRNA microarrays, suggesting that the combinational approach using small RNA sequencing, miRNA microarrays, and computational analysis is effective for identifying miRNAs in cotton in the absence of a complete genome sequence.

An important criterion for miRNA identification is the prediction of secondary structures of potential miRNA hairpin precursors that are recognized and processed by the *Dicer *endonuclease complex [17,18]. Without a sequenced genome in cotton, it is difficult to map potential novel miRNAs to the cotton genome sequences and predict the potential precursor secondary structures based on 300 bp upstream and downstream of the MIR locus. The analysis of cotton ESTs provided a small fraction of potential miRNA precursors (Additional data file 2), which is consistent with results from a recent study in which only eight conserved miRNA loci were identified from ESTs [28]. In addition to EST precursors, putative precursord for *GhMIR164 *and *GhMIR167 *were cloned by genomic walking and characterized (Additional data file 2).

Many canonical miRNAs are conserved among moss, eudicots, and monocots, and some have conserved functions among land plants [36]. For example, the mature canonical miR167 in cotton is identical to that in poplar but has a one-nucleotide substitution relative to that in *Arabidopsis*. The 5' and 3' ends of miR165 are conserved among the canonical sequences in *Arabidopsis*, cotton and poplar (Figure 5) but show three to four nucleotide changes in the middle, which may affect target specificity. The high degree of sequence conservation among miRNAs may explain why miRNA probes designed from different species such as rice, corn, and soybean can cross-hybridize with cotton miRNAs in the microarray analysis. However, data obtained using a heterologous microarray system should be cautiously interpreted and experimentally validated because sequence variation in some miRNAs may cause hybridization differences. Except for a few miRNAs, the identification of cotton miRNAs in this study is based on two or more methods, including miRNA sequence homology, precursor prediction, microarray expression, RNA blot analysis, target identification, and identification of miRNA* (Table 3). The conserved miRNAs may play important roles in cotton fiber and ovule development, as many predicted targets mediate biological pathways such as auxin response and cell patterning that previous studies have implicated in regulating cotton fiber development [6].

Four novel miRNA families, including Gh-miR2948 (miR482-5p), are particularly interesting because they are predicted to mediate the expression for a new set of genes that may affect cotton ovule and fiber development. Gh-miR2948 is identified in cotton but absent in *Arabidopsis *and poplar. One of its predicted targets (ES815756) encodes sucrose synthase, and another predicted target (ES798923) encodes a glucose-methanol-choline oxidoreductase family protein. Sucrose synthases are known to affect fiber cell initiation, elongation, and seed development [37]. Suppression of sucrose synthase (*SUS3*, U73588) expression results in fiberless phenotypes and shrunken or collapsed ovules and seeds. Moreover, the level of *sus3 *suppression is associated with the degree of inhibition of fiber initiation and elongation. The genes encoding glucose-methanol-choline oxidoreductase family protein are expressed in the developing roots and shoots, but their functions are not well understood. These data suggest that Gh-miR2948 has a potential role in the rapid and dynamic process of fiber cell initiation and elongation.

One of the Gh-miR2947 targets encodes a putative serine/threonine protein phosphatase 7. Gh-miR2949 is predicted to originate from three ESTs that are probably derived from homoeologous loci in allotetraploid cotton and related progenitors. For example, two precursors of cotton miR165 are derived from *G. hirsutum *and *G. raimondii*, respectively (Figure 5). Interestingly, the targets of Gh-miR2950 include a gene encoding gibberellin 3-hydroxylase 1, which controls internode elongation in pea, originally described by Gregor Mendel [53]. A naturally occurring mutation in an orthologous gene in *M. sativa *is associated with a dwarf growth phenotype [54]. It is likely that Gh-miR2950 may affect fiber cell elongation.

Gh-miRcand1 has abundant sequence reads and is detected by small RNA blots in ovules and fibers but does not have predicted precursors from available cotton ESTs. Gh-miRcand1 guides infrequent degradation for a predicted target (TC121013) encoding a putative NAC domain transcription factor. This suggests that origin and function of novel miRNAs, including this candidate new miRNA, need further investigation after the cotton genomes are sequenced.

### Down-regulation of miRNAs and its functional implications during early stages of fiber development

Normalized miRNA reads, miRNA microarrays, and miRNA blot analysis collectively show that many miRNAs accumulate at lower levels in fibers (10 DPA) and fiber-bearing ovules (3 DPA) than immature ovules 3 days prior to anthesis. Several conserved miRNAs, including cotton miR156, miR159, miR165, miR166, miR167, miR168, miR171, and miR172, were expressed at high levels in the immature ovules (-3 DPA) but at relatively low levels in fiber-bearing ovules and fibers. The low levels of miRNA accumulation in the fibers and fiber-bearing ovules (Figure 4) may allow target gene expression that is required for fiber cell development, which is consistent with upregulation of several targets of miRNAs, including three novel miRNAs tested (Figure 6). Moreover, the ESTs encoding putative phytohormone regulators and transcription factors were generally upregulated during early stages of fiber development [3,7,55,56]. Thus, miRNA regulation may be important to fine-tune developmental and metabolic pathways during early stages of fiber cell initiation and elongation. These miRNAs may serves as negative regulators for cotton fiber development if their targets play a positive role. Alternatively, these miRNAs may positively affect cotton fiber development if their targets are negative regulators.

Gh-miR168 is involved in feedback regulation of miRNA biogenesis because its target *AGO1 *is essential for miRNA biogenesis and for miRNA-guided mRNA degradation [57]. A low level of miR168 expression in the immature ovules at -3 DPA may result in a high level of *AGO1*, suggesting an active role of miRNAs during early stages of fiber cell initiation and ovule development. Two Gh-miR167 targets encode ARF6 and ARF8. In *Arabidopsis*, miR167 controls *ARF6 *and *ARF8 *expression patterns and affects the fertility of ovules and anthers [58] probably because Gh-miR167 directs cleavage of targets that are negative regulators of an auxin response pathway [58,59]. *OsGH3-2 *is an auxin responsive transcript that encodes an IAA-conjugating enzyme in rice, and *OsGH3-2 *expression is regulated by miR167-mediated *ARF8 *expression [59]. Overexpression of mutagenized miR167 targets *ARF6 *and *ARF8 *in *Arabidopsis *leads to sterility in ovules and anthers [58]. Like Gh-miR167, the novel Gh-miR2950 was poorly expressed in fibers (+7 DPA), and two of its putative targets encoded putative gibberellin 3-hydroxylase 1 in cotton, suggesting a role for gibberellins in ovule and fiber cell development.

miR165/166 plays a critical role in shoot apical meristem initiation [60] and leaf polarity and pattern formation [61] in *Arabidopsis*. The relatively high level of Gh-miR165/166 in immature ovules (-3 DPA) relative to fiber-bearing ovules (+ 3 DPA) may suggest a role for Gh-miR165/166 in fiber cell initiation, a process in some ways similar to shoot apical meristem initiation. Interestingly, the expression levels of these two miRNAs in fiber-bearing ovules (0 and +3 DPA) are higher in the *N1N1 *lintless mutants than in the wild type, suggesting negative effects of this miRNA on fiber cell development. The predicted targets of Gh-miR165/166 include a dozen ESTs encoding the class III HD-Zip proteins (Additional data file 3), many of which are differentially expressed during fiber development (data not show), suggesting that these targets are repressed by miR165/166 during the transition from immature ovules to rapid fiber initiation and development in cotton. In *Arabidopsis*, class III HD-Zip proteins are required for the formation of a functional shoot apical meristem [62]. Gh-miR165/166 guides cleavage of three HD-Zip protein 5 and 8 genes examined (Figure 5), but is not obviously correlated with target gene expression levels (Figure 6). This suggests that these targets are affected by other mechanisms, such as translational repression. Alternatively, they may not be the main targets of miR165/166 in cotton.

Auxin is a positive regulator of fiber initiation during ovule culture *in vitro *[2]. Gh-miR167 is expressed at higher levels in immature ovules (-3 DPA) and fiber-bearing ovules (0 and +3 DPA) than in isolated fibers (7 DPA) in *G. hirsutum*. Gh-miR167 is also expressed in the ovules (+ 3 DPA) of *G. raimondii *and the *N1N1 *mutant, which develop ovules and seeds but do not produce lint fibers. Seven ESTs are predicted to be Gh-miR167 targets encoding ARFs, several of which are targeted for degradation by Gh-miR167 (Figure 5). ARFs regulate fruit initiation in *Arabidopsis *and tomato. A mutation in *ARF8 *results in the separation of fruit development from fertilization and produces seedless (parthenocarpic) fruit in *Arabidopsis *[63]. In tomato, *ARF7 *is expressed at a constantly high level in mature flowers and repressed within 2 days after pollination. Transgenic plants with decreased *SlARF7 *mRNA levels bear seedless fruits [64]. The data suggest that Gh-miR167 regulates ovule development and seed formation and probably indirectly affects fiber development.

The difference in miR164 accumulation between fiber-bearing ovules in the tetraploids and fiber-poor ovules in *G. thurberi *and *N1N1 *mutant indicate that miR164 plays a role not only in early stages of fiber development but also in organ (ovule) formation in diploid and tetraploid species. In *Arabidopsis*, miR164 regulates the expression of *CUP-SHAPED COTYLEDON1 *(*CUC1*) and *CUC2*, and overexpression of mutagenized *CUC1 *mRNA induces alterations in embryonic, vegetative, and floral development [65]. The family of NAM/CUC transcription factors functions downstream of transport inhibitor response 1 (TIR1) in response to auxin, which is down-regulated by miRNAs. Accumulation of miR164 in elongating fibers suggests roles for auxin regulation and NAM-like genes in ovule and fiber development.

In *Arabidopsis*, miR390 along with AGO7 acts in *TAS3 *tasiRNA biogenesis, allowing TAS3 regulation of *ARF3 *expression [40]. *ARF3 *and *ARF4 *function in organ asymmetry, and TAS3 tasiRNAs mediate regulation of these *ARF *genes, which affect leaf morphology, developmental timing and patterning [39,40]. miR390a is expressed at high levels during early stages of fiber development but at low levels in the leaves or fibers, suggesting potential roles for miR390 and TAS3 in ovule and fiber development through *ARF3 *and *ARF4 *regulation.

Auxin promotes the degradation of the Aux/IAA transcriptional repressors by stimulating Aux/IAA binding to the auxin receptor F-box proteins, including TIR1, which brings Aux/IAA proteins to the SCF ubiquitin ligase complex for ubiquitylation and subsequent proteasomal destruction [66]. miR393 is predicted to target *TIR1 *(Additional data file 3) and is expressed at higher levels in ovules (+3 DPA) and fibers (+7 DPA) than in ovules of the *N1N1 *mutant, suggesting that *TIR1 *regulation is important for fiber cell development. Upregulation of miR393 may lead to miRNA-triggered degradation of *TIR1*. Further analysis of *TIR1 *family mRNA and protein levels in cotton ovules with altered miR393 expression or expression of mutagenized *TIR1 *mRNA will help in understanding the functions of miR393 and *TIR1 *in fiber cell development.

MYB transcription factors mediate trichome development in leaves and fiber development in cotton [8-10]. A total of six ESTs encoding putative MYB family proteins are predicted to be the targets of several moderately and lowly conserved miRNAs, including cotton miR399 and miR828 (Additional data file 3). Although the exact functions of these miRNAs and specific cotton MYB transcription factor genes are unknown, cotton GaMYB2 is a homolog of GL1, which plays a role in fiber development [9]. Among 198 genes encoding MYB transcription factors in *Arabidopsis*, only a few are predicted to be the miRNA targets (by miR159 and miR828). Two miR828 targets are *MYB82 *and *MYB113 *in *Arabidopsis*. In cotton, Gh-miR828 is predicted to target five ESTs encoding putative MYB transcription factors and one EST encoding ARABIDOPSIS THALIANA KINESIN 3 (ATK3). Kinesin-like proteins are known to affect cellulose microfibrils and cell wall strength [67]. In general, the number of targets predicted in cotton was relatively large, probably because many EST tentative consensus (TC) sequences may contain paralogous and homoeologous transcripts derived from two progenitors' genomes in allopolyploid cotton. Alternatively, the number of ESTs may be artificially enlarged because of computational limitations in accurately predicting paralogous and homoeologous ESTs. Collectively, high levels of miRNA accumulation during early stages of fiber cell initiation and elongation may temporarily down-regulate some physiological pathways prior to fiber cell initiation. Towards fiber elongation, the accumulation levels of many miRNAs are decreased, which may promote signaling and metabolic pathways such as auxin and gibberellin signaling, cell patterning, and sucrose and cellulose biosynthesis that are essential for fiber cell initiation and elongation as well as ovule development.

## Conclusions

Analyses of massively parallel sequencing data, miRNA microarrays, small RNA blots, and target cleavage assays indicate that rapid and dynamic changes in gene expression and physiology are correlated with a general enrichment of 24-nucleotide siRNAs and repression of many miRNAs during ovule and fiber development in allotetraploid cotton. siRNAs are derived from a small proportion of large cotton EST collections but match relatively large amounts of WGS trace reads of the *G. raimondii *genome. The enrichment of siRNAs in ovules and fibers relative to non-fiber tissues, including leaves, suggests active small RNA metabolism and chromatin modifications during fiber development. The general repression of miRNAs, including novel miRNAs, in fibers correlates with upregulation of a dozen validated miRNA targets encoding transcription and phytohormone response factors, including genes such as *ARFs *and *SUS3 *that are found to be highly expressed in cotton fibers. This work provides a rich source of genomic and gene expression data and several new findings, including 4 novel miRNAs, one candidate-novel miRNA, 25 new miRNA precursors, and over 200 predicted targets, which will facilitate future studies on the role of miRNAs and siRNAs in cotton fiber development.

## Materials and methods

### Plant materials and growth conditions

Wild-type *G. hirsutum *L. cv. Texas Marker-1 (TM1), the near-isogenic *N1N1 *mutant in TM1, *G. arboretum *(A2), and *G. thurberi *(D1) were grown in a greenhouse at 30 to 35°C under ambient light supplemented with continuous fluorescent illumination. Bolls were tagged on the day of anthesis (0 DPA), and the stages of pre-anthesis flowers (for example, three days prior to anthesis, - 3 DPA) were estimated based on flower bud size and shape. Harvested bolls and leaves were stored in ice until dissection, and the ovules were carefully dissected from each boll, frozen in liquid nitrogen, and stored at -80°C.

### Cotton small RNA sequencing

Total RNA in different cotton tissues was extracted using a modified CTAB protocol [68] excluding polyvinyl pyrrolidone in the extraction buffer, which was found to cause RNA degradation specifically with *G. thurberi *leaf extractions and was dispensable for cotton RNA isolation (data not shown). LiCl precipitation may enrich large RNAs [69,70], but this did not seem to affect the size distribution of small RNAs in sequencing and small RNA blot analyses (see Results). RNA concentration was estimated using a Nanodrop spectrophotometer, and RNA quality was examined using denaturing formaldehyde agarose gel electrophoresis. An aliquot of total RNA (75 μg) each prepared from ovules (-3, 0, 3 DPA) and leaves was run on 15% denaturing polyacrylamide gel (7 M urea) with RNA size markers. The small RNAs of 17 to 27 nucleotides were purified in a 15% polyacrylamide gel and ligated to the 5' RNA adaptors, resulting in 37- to 47-nucleotide RNAs. After the ligation with both 5' and 3' RNA adaptors, the RNA bands ranged from 57 to 87 nucleotides. After purification, the ligated RNAs from each sample were reverse-transcribed using primer pairs partially corresponding to the RNA adaptors. The first-stranded cDNAs in each sample were amplified using the primer pair that contains a specific nucleotide as a 'barcode.' The four 'barcoded' samples were pooled in equal amounts, and a small aliquot of pooled DNA was cloned and sequenced to determine the quality and representation of cloned products. After the quality control was completed, the pooled DNA was subjected to high-throughput sequencing using an Illumina G1 Genome Analyzer. A total of approximately 4 million reads was generated.

### Sequence analysis of small RNAs, miRNAs and their targets

Cellular RNAs were annotated based on homology to: *A. thaliana *and *Brassica napus *mitochondrial DNA; *G. hirsutum *plastid DNA; plant small nuclear RNAs (snRNAs) and small nucleolar RNAs (snoRNAs) in NCBI; *A. thaliana *tRNA sequences; and *A. thaliana *and *G. hirsutum *rRNA. These 'contaminant' sequences were grouped as raw sequence reads by their barcoded bases and adaptor sequences. Reads with and without contaminant sequences were then mapped onto the TIGR CGI9 EST assembly and also onto WGS trace reads from 0.5× of the *G. raimondii *genome (Table 1). The small RNA read libraries might contain sequencing errors because the *G. hirsutum *genome sequence is not available.

Conserved miRNAs were identified based on homology with less than two mismatches to known miRNAs deposited in miRBase 13.0 [71]. After removal of other cellular RNA sequences, miRNA abundance was normalized to TPQ. The most abundant miRNA variant was used as a query sequence to search for potential targets using methods as described [19,21] (Additional data file 3). To annotate the potential target ESTs, a BlastX search was performed against the NCBI non-redundant protein database of flowering plants (taxid: 3398) and the top three hits (*P *= 0.001) were chosen for the potential function of each query [72]. The ESTs exist in the sense or antisense orientations, and the candidate targets with an open reading frame in the same 5' to 3' directionality as that of the miRNA were removed. However, a few partial ESTs likely derived from miRNA precursors in ambiguous orientations might still remain among predicted targets.

Pre-miRNA precursors were searched against CGI9 using MIRcheck [19,36] with a 600-nucleotide window centered on a cotton small RNA sequence (Additional data file 2). Known miRNA families were annotated based on conservation of hairpin-loop structures as well as mature miRNA sequences, while a novel miRNA precursor was identified only if a miRNA* sequence was also detected on the opposite strand of the hairpin [34]. Hairpin stem-loop structures were analyzed and visualized using the sir graph tool in the UNAFold package [35].

### Accession numbers for small RNA sequences, miRNA microarrays, and miRNA genomic precursors

All predicted miRNAs were deposited in miRBase. Small RNA sequencing data and miRNA microarray expression data reported in this paper were deposited in the NCBI Gene Expression Omnibus database under the series record numbers [GEO:GSE16632] ([GEO:GSM409656-GSM409659]) and [GEO:GSE16986], respectively. GenBank accession numbers of cotton genomic sequences for two miRNA precursors are [Genbank:GU190712] for *Gh-MIR164 *and [Genbank:GU190713] for *Gh-MIR167*.

### Conserved miRNA expression detected by miRNA microarrays

miRNA microarrays were performed using the arrays manufactured by CombiMatrix (Mukilteo, WA, USA) [38]. Small RNAs from leaves, fibers (7 DPA), fiber-ovules (3 DPA) of TM-1, and ovules (+3 DPA) of the near-isogenic *N1N1 *mutant were enriched from total RNA using a mirVana isolation kit (Ambion, Austin, TX, USA). Each custom-designed chip contained four identical arrays spotted with anti-sense DNA oligonucleotides corresponding to miRNAs (version 9.1) [71], tasiRNAs, and selected endogenous siRNAs from the *Arabidopsis *Small RNA Project [73]. After prehybridization, the chip was hybridized to small RNAs labeled with Cy5 (Mirus Bio, Madison, WI, USA) overnight at 37°C. Posthybridization washes were performed sequentially, once each with 6× SSPE (900 mM NaCl, 60 mM NaH_2_PO_4_, 6 mM EDTA, pH 7.4) and 3× SSPE and twice with 0.5× SSPE (each solution containing 0.05% Triton X-100). Finally, hybridized chips were scanned using a Genepix 4000B (Molecular Devices, Sunnyvale, CA, USA), and data were extracted using software provided by CombiMatrix. A total of 12 hybridization intensities in three biological replicates per miRNA were obtained, and log values of hybridization intensities were used for normalization and statistical analysis. If the hybridization intensities of a miRNA were not significantly higher than the background signals (Student's *t*-test, *P *= 10^-4^), the miRNA was considered to be not expressed.

The statistical analysis was performed as previously described [74]. Briefly, we used a linear model to examine significance of different miRNA accumulation levels in different developmental stages and mutant lines and exclude effects of technical and biological variation. The linear model for expression intensity of miRNA (G)_i _in replicate (R)_j _and stages (S)_k _is as follows:

where i = 1... 117; j = 1, 2, 3; k = 1, 2, 3, 4; and G, R, and S are main sources of variation from gene (G), replicate (R), and stages or conditions (S).

The null hypothesis (H_o_): S_k _+ (GS)_ik _= S_k_' + (GS)_i_'_k_', was used to test the miRNA difference between two stages (for example, TM-1 leaves and ovules at +3 DPA). Differential accumulation of miRNAs among TM-1 fiber (+7 DPA), *N1N1 *mutant ovules (+3 DPA), TM-1 fiber-bearing ovules (+3 DPA), and TM-1 leaves was tested using F-test in the linear model for distinct mature miRNAs:

The type I error rate of 117 tests was adjusted using the false discovery rate (α = 0.05) of Benjamini and Hotchberg [75].

### miRNA Northern analyses

Total RNA (25 μg) per sample was resolved in a 15% denaturing polyacrylamide gel (8 M Urea; (National Diagnostics, Atlanta, GA, USA) and was transferred to Amersham Hybond™-N+ nylon membranes (GE Healthcare, Piscataway, NJ, USA). DNA oligonucleotide probes (Additional data file 5) were labeled with γ^32^P-ATP using T4 Polynucleotide kinase (Promega, Madison, WI, USA). The blot was prehybridized in Church buffer [76] for 2 hours at 37°C. After hybridization with a miRNA probe overnight, the blot was washed twice with a low-stringency buffer (1× SSC, 0.5% SDS) at 37°C for 15 minutes followed by a high-stringency buffer wash (0.2× SSC, 0.2% SDS) at 37°C for 15 minutes 1 to 3 times, depending on the background counts. The blot was then sealed in a plastic bag and exposed to a PhosphorImager screen (Fuji) for 3 hours to overnight at room temperature. The screen was scanned using a Molecular Imager FX (Bio-Rad, Hercules, CA, USA), and the images were processed using ImageQuant software (GE Healthcare, Piscataway, NJ, USA). The blots were stripped off the probe in a boiling solution containing 0.5% SDS for approximately 10 minutes and reprobed using another anti-sense miRNA probe.

### RNA ligase-mediated 5' RACE

To map the cleavage sites of target transcripts, we used RNA ligation-mediated (RLM) rapid amplification of 5' complementary DNA ends (5' RACE) using a GeneRacer kit (Invitrogen, Carlsbad, CA, USA) that was modified from a published protocol [41]. In brief, total RNAs (4 μg) from equal mixtures of ovules (-3, 0, +3 DPA) and fibers (10 DPA) were ligated to a 5' RACE RNA adapter without calf intestine alkaline phosphatase treatment. cDNAs were transcribed with reverse transcriptase and the GeneRacer Oligo dT primer. A pool of non-gene-specific 5' RACE products was generated using the GeneRacer 5' Primer (5'-CGACTGGAGCACGAGGACACTGA-3') and the GeneRacer 3' Primer (5'-GCTGTCAACGATACGCTACGTAACG-3'). Gene-specific 5' RACE amplifications were conducted with the GeneRacer 5' Nested Primer and gene-specific primers listed in Additional data file 5. The 5' RACE amplification products were gel purified and cloned, and 15 to 50 inserts were cloned and sequenced from a typical reaction.

### Quantitative RT-PCR analysis

Gene-specific primers were designed using Primer Express version 2.0 software (Applied Biosystems, Foster City, CA, USA; Additional data file 5). The qRT-PCR reaction was carried out in a final volume of 20 μl containing 10 μl SYBR Green PCR master mix, 1 μM forward and reverse primers, and 0.1 μM cDNA probe in a ABI7500 Real-Time PCR system (Applied Biosystems). Cotton *HISTONE H3 *(AF024716) was used to normalize the amount of gene-specific RT-PCR products [9]. All reactions were performed in three replications using a dissociation curve to control the absence of primer dimers in the reactions. The amplification data were analyzed using ABI7500 SDS software (version 1.2.2), and the relative expression levels (fold changes) were calculated using the standard in each reaction.

## Abbreviations

AGO: Argonaute; At: *Arabidopsis thaliana*; ARF: auxin response factor; Aux: auxin; CGI: Cotton Gene Index; DCL: Dicer-like; DPA: days pre-/post-anthesis; EST: expressed sequence tag; Gh: *Gossypium hirsutum*; Gm: *Glycine max*; IAA: indole acetic acid; miRNA: microRNA; Mt: *Medicago truncatula*; NAM: no apical meristem; Os: *Oryza sativa*; Pol: polymerase; Pp: *Physcomitrella patens*; Pt: *Populus trichocarpa*; qRT-PCR, quantitative RT-PCR; RACE: rapid amplification of complementary DNA ends; rasiRNA: repeat associated siRNA; Sb: *Sorghum bicolor*; So: *Saccharum officinarum*; siRNA: small interfering RNA; tasiRNA: *trans*-acting siRNA; TIR1: transport inhibitor response 1; TM-1: Texas Marker-1; TPQ: transcripts per quarter million; WGS: whole-genome shotgun; Zm: *Zea mays*.

## Authors' contributions

ZJC designed experiments, PM performed miRNA expression assays and validated miRNA targets, AWW made small RNA libraries and performed initial sequence analysis, VA analyzed small RNA sequence data and predicted miRNA targets, VR performed miRNA microarray experiments, MH analyzed mRNA microarray data, XG performed miRNA target gene expression assays, XC analyzed miRNA data, BAT and DMS contributed plant materials, and PM, VA, AWW and ZJC analyzed data and wrote the paper.

## Additional data files

The following additional data are available with the online version of this paper: a table listing normalized small RNA reads (transcripts per quarter million) per megabase of available repetitive sequences (Additional data file 1); a table listing cotton miRNAs and their predicted precursors (Additional data file 2); a table listing cotton miRNAs and their targets predicted from ESTs (Additional data file 3); a table listing differentially expressed miRNAs in cotton (Additional data file 4); a table listing the primers for target cleavage assays and oligonucleotide probes for small RNA blot analysis (Additional data file 5).

## Supplementary Material

Additional data file 1 Normalized small RNA reads (transcripts per quarter million) per megabase of available repetitive sequences. Click here for file

Additional data file 2 Cotton miRNAs and their predicted precursors. Click here for file

Additional data file 3 Cotton miRNAs and their targets predicted from ESTs. Click here for file

Additional data file 4 Differentially expressed miRNAs in cotton. Click here for file

Additional data file 5 Primers for target cleavage assays and oligonucleotide probes for small RNA blot analysis. Click here for file
